# Mechanisms, functions and ecology of colour vision in the honeybee

**DOI:** 10.1007/s00359-014-0915-1

**Published:** 2014-05-15

**Authors:** N. Hempel de Ibarra, M. Vorobyev, R. Menzel

**Affiliations:** 1 Department of Psychology, Centre for Research in Animal Behaviour, University of Exeter, Exeter, UK; 2 Department of Optometry and Vision Science, University of Auckland, Auckland, New Zealand; 3 Institute of Biology - Neurobiology, Freie Universität Berlin, Berlin, Germany

**Keywords:** Honeybee, Colour vision, Models of colour discrimination, Colour learning, Insect behaviour

## Abstract

Research in the honeybee has laid the foundations for our understanding of insect colour vision. The trichromatic colour vision of honeybees shares fundamental properties with primate and human colour perception, such as colour constancy, colour opponency, segregation of colour and brightness coding. Laborious efforts to reconstruct the colour vision pathway in the honeybee have provided detailed descriptions of neural connectivity and the properties of photoreceptors and interneurons in the optic lobes of the bee brain. The modelling of colour perception advanced with the establishment of colour discrimination models that were based on experimental data, the Colour-Opponent Coding and Receptor Noise-Limited models, which are important tools for the quantitative assessment of bee colour vision and colour-guided behaviours. Major insights into the visual ecology of bees have been gained combining behavioural experiments and quantitative modelling, and asking how bee vision has influenced the evolution of flower colours and patterns. Recently research has focussed on the discrimination and categorisation of coloured patterns, colourful scenes and various other groupings of coloured stimuli, highlighting the bees’ behavioural flexibility. The identification of perceptual mechanisms remains of fundamental importance for the interpretation of their learning strategies and performance in diverse experimental tasks.

## Introduction

Colour vision of the honeybee, *Apis mellifera* L., has been studied in more detail than that of any other animal apart from primates. Furthermore, the honeybee was the first non-human animal for which colour vision was convincingly demonstrated. Lubbock (1882) reported that foraging honeybees repeatedly visited coloured cards when rewarded with drops of honey. Trained and recruited bees quickly learnt to distinguish a rewarded colour from several alternatives. Further observations of colour discrimination and wavelength-dependent preferences followed in other animals, such as water flees and fish (e.g. Lubbock 1888; von Frisch 1912), but the experiments by von Frisch (1914) with honeybees were the most significant ones proving the existence of colour vision in non-human animals. Von Frisch (1914) first trained bees to a coloured card by rewarding them with sucrose solution. Subsequently, in unrewarded tests the coloured card was presented together with grey cards of different intensities (initially 30 shades of grey, later reduced to 15). He reasoned that if an animal relied on the intensity of a stimulus one of the cards would match subjective intensity of a coloured stimulus and the animal would not be able to discriminate a particular shade of grey from colour. This grey-card experiment, originally proposed by the ant researcher Forel a few years earlier (von Frisch 1914), is now considered to be a classic behavioural paradigm for demonstrating colour vision in animals, and has been successfully applied to many animal species (for an overview see Kelber et al. 2003).

These behavioural studies of colour vision in honeybees were extended by Lotmar (1933) and Mazokhin-Porshnyakov (1969), who varied the range of tested visual stimuli and reward schemes. Their experiments confirmed von Frisch’s finding that bees in such choice experiments were guided by the colour rather than the brightness of stimuli. Kühn (1924) determined that the spectral range of bees’ vision includes ultraviolet (UV), a feature that they share with many other animals (for a review see Tovée 1995). Kühn (1924) further suggested that bees discriminate colours best if they originated from different parts of the visible spectrum separated by approximately 80–100 nm. Later he found effects that resembled the simultaneous colour contrast known from human colour perception, and suggested that some colours could form complementary colour pairs (Kühn 1927). These findings motivated Daumer (1956) to design carefully controlled colour-mixing experiments for determining the dimensionality of bee colour vision. Daumer (1956) based his study on the rationale of psychophysical experiments in human vision research. He mixed monochromatic lights from two different wavelength ranges (e.g. 360 and 490 nm), hypothesising that they would be complementary colours if not distinguished against a white stimulus (including the UV range, i.e. UV-white), which should appear colourless to bees. Furthermore, he found that a mixture of two lights from different parts of the spectrum (appearing to humans as blue and yellow) could be matched to a metameric intermediate colour that was undistinguishable for bees. He identified three primary colours in the short-, middle- and long-wavelength regions and concluded that bees have trichromatic colour vision. Daumer (1956) also demonstrated that bees perceive bee-subjective purple, which results from the joint stimulation with light at the short- and long-wavelength end of the visible spectrum.

The hypothesis of honeybee trichromacy was later confirmed by intracellular recordings from photoreceptor cells (Autrum and von Zwehl 1964; Menzel 1975; Menzel and Blakers 1976) demonstrating that bees have three spectral types of photoreceptors peaking in UV, blue and green parts of the spectrum. The peak sensitivities of the three photoreceptor types lie within the wavelength range of the spectral primaries determined by Daumer’s colour-mixing experiments (Daumer 1956). All three receptor types contribute to trichromatic colour vision. Thus, in the case of bees the number of retinal spectral units corresponds to the dimensionality of colour vision, a relationship that does not always hold true in animal vision, where not all photoreceptor classes are involved in colour vision (e.g. Koshitaka et al. 2008). Overall, the work conducted up to the 1970s contributed major insights establishing that bee colour vision shares basic similarities with human and primate colour vision, despite having evolved in an invertebrate and having a visual range that is shifted towards shorter wavelengths.

**Fig. 1:**
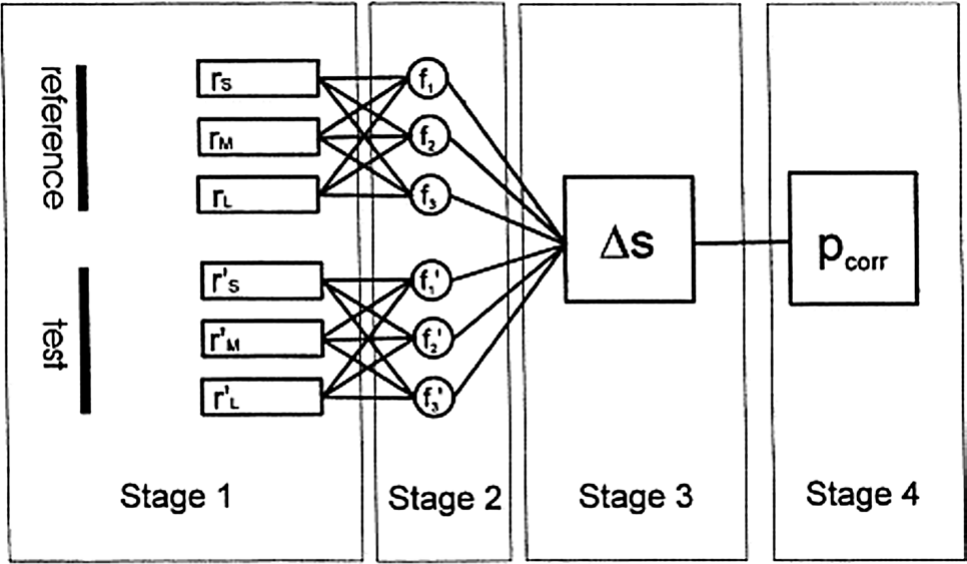
Schematic diagram of the four functional stages in the process of colour discrimination. *Stage 1* receptor signals arise from quantum absorption (*r*) in the S, M and L-receptors for each of two different stimuli (reference and test stimulus). Quantum catches are transduced into graded voltage signals giving rise to receptor signals: {*r*_S_ *r*_M_ *r*_L_} for the reference stimulus and (*r*′_S_ *r*′_M_ *r*′_L_) for the alternative test stimulus. This stage may also include any postreceptoral processing if the segregation between different receptor types is preserved. *Stage 2* neural coding of the receptor signals into achromatic and/or chromatic signals. Three coding units are depicted, the output of which is given by *f*_1_ *f*_2_ *f*_3_ and *f*′_1_ *f*′_2_ *f*′_3_, for the reference and test stimulus, respectively. The units depicted here by a single symbol may represent several morphological and/or physiological layers. *Stage 3* the two sets of signals are compared and evaluated. The output of this stage is the computed difference between *stage 2* signals using a particular algorithm. Metric models will calculate a perceptual distance ΔS for the stimulus pair. *Stage 4* the behavioural response, which is represented by the probability *p*_corr_ that one of two stimuli is selected. According to the metric approach *p*_corr_ depends on ΔS alone. Reprinted from Brandt and Vorobyev (1997), with permission from Elsevier

Subsequent efforts were directed towards uncovering the basic mechanisms of colour coding, capitalising on evidence emerging from neurophysiological studies, mathematical modelling, and advances in technology allowing effective control of stimuli and measurement of spectra (for reviews see Menzel 1979a; Menzel and Backhaus 1991; Kelber et al. 2003). The following scheme illustrates the main rationale that has guided the study of colour vision in honeybees at different levels, from receptors to behaviour (Fig. 1), assuming two main coding principles—trichromacy at the retinal level and colour opponency at the post-receptor neural level (Menzel and Backhaus 1989; Brandt and Vorobyev 1997). The basic prerequisite for colour vision is the existence of photoreceptors with different spectral sensitivities. In the first stage of colour coding, three types of bee photoreceptors with different spectral sensitivity absorb light quanta in specific wavelength ranges. The second stage corresponds to neural post-receptor mechanisms where receptor signals are processed and subtracted. Although shown as a single layer with three coding units, their number might vary and they could be distributed across several successive morphological and physiological layers. In the third stage, outputs of post-receptor mechanisms are compared between stimuli. A result of this comparison is the estimate of similarity between the stimuli. The fourth stage is the behavioural response, which depends on the result of the similarity estimate. The relationship between the first input stage and the final behavioural output has been thoroughly characterised, and models have provided useful quantitative descriptions, as discussed further below. It has, nevertheless, been difficult to identify the elements and functional connectivity of the neural circuitry involved in colour coding.

## Neural substrates of colour vision in bees

The honeybee eye contains three types of photoreceptors which peak in the UV, blue, and green parts of the spectrum: S (short-wavelength sensitive, *λ*_max_ = 344 nm), M (middle-wavelength sensitive, *λ*_max_ = 436 nm), and L (long-wavelength sensitive; *λ*_max_ = 544 nm) receptors, respectively (Menzel and Backhaus 1991, Fig. 2). Receptor signals contribute to primary post-receptor mechanisms that code spectral information. These mechanisms may be of two kinds—colour-opponent (subtractive) mechanisms code chromatic aspects of coloured stimuli; and non-opponent mechanisms code achromatic aspects, such as brightness. Non-opponent mechanisms may either sum inputs from several receptor types or use input from just one type of receptor. Chromatic mechanisms are sensitive to changes in the spectral composition of light stimuli, while achromatic mechanisms are sensitive to changes in intensity. In the honeybee all three types of photoreceptors contribute to colour coding, whereas brightness coding is mediated predominantly by the L-receptor (e.g. Srinivasan and Lehrer 1984, 1988; Giurfa et al. 1996). The extent to which the S- and M-receptor contribute to achromatic vision has not been convincingly established, but some experimental studies indicate the possible involvement of the M-receptor (Menzel 1981; Zhang et al. 1995; Giurfa et al. 1999). Also, phototactic responses can be elicited with wavelengths across the visible spectrum of bees and it has been argued that phototaxis is based on the integration of signals from all three receptor types (Kaiser et al. 1977; Menzel and Greggers 1985). However since the L-receptor is sensitive in blue and even UV parts of the spectrum, it is possible that achromatic sensitivity is determined by the L-receptor in all contexts.

**Fig. 2:**
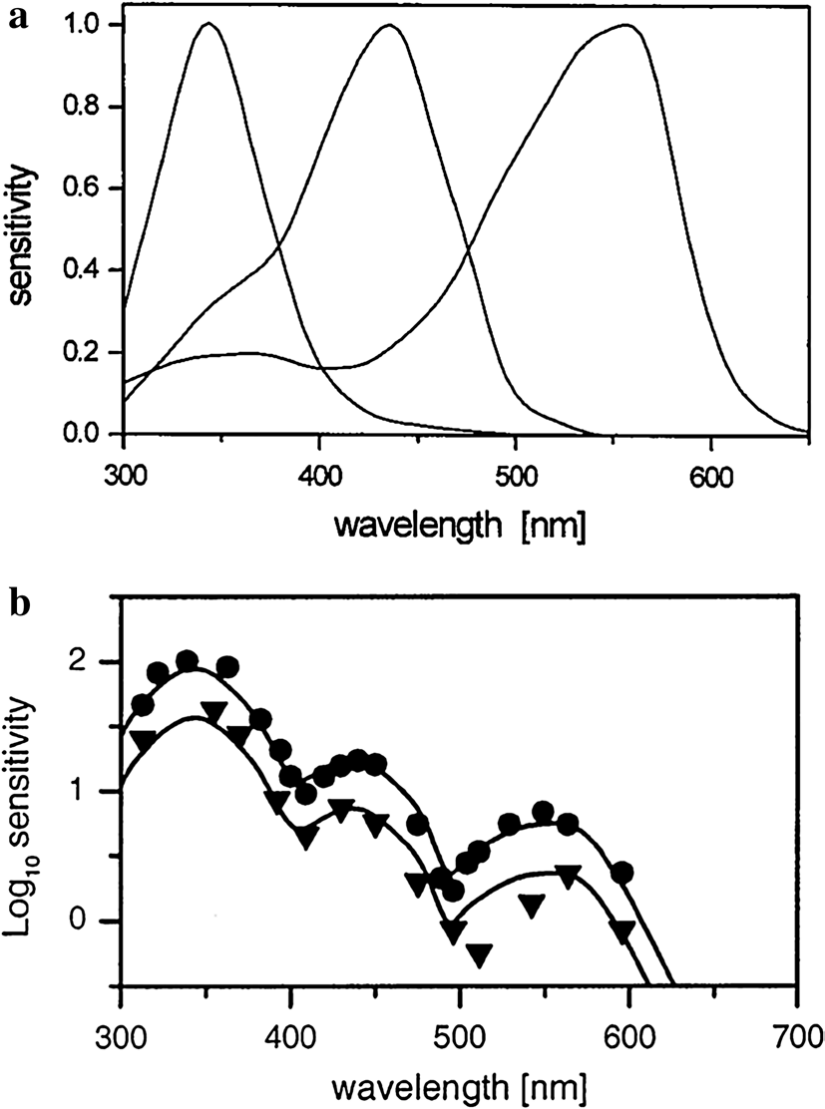
Spectral sensitivity of the honeybee. **a** Spectral sensitivity curves of the three photoreceptor types of the honeybee (Menzel and Backhaus 1991). The functions are scaled to the maximal sensitivity of each receptor at their peak wavelength (S—344 nm, M—436 nm, L—556 nm). **b** Behavioural threshold spectral sensitivity of the honeybee (*symbols*) and the predicted threshold function by the RNL Colour-Opponent model (*solid curve*, Vorobyev and Osorio 1998). The behavioural data were obtained by von Helversen (1972) with two bees (bee 25—*circles*, bee 15—*triangles*, bee numbers as given in the original paper). Reprinted from Vorobyev et al. (2001a), with permission from Elsevier

Each ommatidium of the bee eye contains eight large photoreceptor cells and one basally located small one. The photosensitive parts of photoreceptor cells, the microvilli, are fused forming a waveguide-like rhabdom along the optical axis of the ommatidium. Early evidence suggested that photoreceptors might not have an even distribution across the retina (Gribakin 1969), but only recently, using in situ hybridization of the three bee opsin mRNAs, three types of ommatidia with different sets of photoreceptors were identified (Wakakuwa et al. 2005) that are unevenly distributed across the eye (less the dorsal rim area specialised in polarised light detection). Each ommatidium contains six large photoreceptors belonging to the L-photoreceptor class (Wakakuwa et al. 2005), but only about 44 % of the ommatidia contain all three receptor classes (Menzel 1975; Menzel and Blakers 1976; Wakakuwa et al. 2005). These are the type I ommatidia, which contain both one S- and one M-receptor. Type II ommatidia (46 %) contain two S-receptors, and only 10 % of ommatidia were identified as Type III with two M-receptors (Wakakuwa et al. 2005). The small ninth photoreceptor was not included in this classification, because it was not possible to reliably label it. Previously, it was thought that the ninth cell is an S-receptor (Menzel and Blakers 1976), or an S- or M-receptor (Gribakin 1972), but Wakakuwa et al. (2005) did not find supportive evidence, and instead suggested that it is more likely to be an L-receptor. In any case, it is unclear whether this cell contributes to colour vision at all. The parallel orientation of the microvilli in its rhabdom suggests a high polarisation sensitivity, and indeed such receptor cells have been recorded (Menzel and Snyder 1974). This could cause undesirable interference with the signals from other receptors that have twisted rhabdoms and thus minimal polarisation sensitivity.

Six photoreceptors from each ommatidium project as short visual fibres into a single cartridge of the lamina, the most distal of three ganglia in the bee optic lobes (Ribi 1975; Ribi and Scheel 1981). Gribakin (1972) illuminated the retina with 480 nm light during osmium fixation for electron microscopy, assuming that it would selectively bleach L-receptors. Six stained photoreceptor axons in the ventral part of the eye projected to the lamina, suggesting that these are L-receptors. However, using intracellular dye marking Menzel and Blakers (1976) found that as well as L-receptors, M-receptors also project to the lamina, which could be another explanation for Gribakin’s finding, considering that M-receptors are also sensitive to 480 nm light. A more recent paper by Friedrich et al. (2011) analysed ommatidial position, morphology and developmental evidence from studies in the honeybee and other hymenopterans, suggesting homologies to *Drosophila* and lepidopterans where the projection patterns of photoreceptors are better known. They conclude, similarly to Gribakin (1972), that the six short visual fibres must be L-receptors.

Three further photoreceptors project their axons through the lamina directly into the medulla. These long visual fibres belong not only to S-receptors (Menzel and Blakers 1976), but also to M-receptors (Friedrich et al. 2011), which are large photoreceptors (Friedrich et al. 2011). Depending on the ommatidial type each the S- or M-receptor or both represent two of the three long visual fibres. The third long visual fibre in each ommatidium is the axonal projection of the small ninth photoreceptor (Ribi 1975). Since it is now widely accepted that the honeybee retina has a heterogeneous ommatidial structure, it would be desirable to finally clarify the spectral type of the ninth cell, to understand its function in visual processing.

The ommatidial projection patterns are retinotopic in both the lamina and medulla. In these two layers of the optic lobes as well as in the third optical neuropile, the lobula (located between the medulla and the protocerebrum), spectrally opponent interneurons that respond differentially to spectral lights across the visible range of the bee have been investigated using intracellular recordings (Menzel 1974; Kien and Menzel 1977a, b; Hertel 1980; Hertel and Maronde 1987; Riehle 1981; Maronde 1991; Yang et al. 2004). It is very difficult to record intracellularly from visual interneurons because they are very small and often difficult to access (for example, in the lamina). Recordings can be maintained only for short periods. Nevertheless, several important features of colour-coding neurons have been described.

Response patterns of the large monopolar cells indicate that colour-opponent processing may occur in the lamina (Menzel 1974; de Souza et al. 1992), and it has been suggested that their outputs could be combined with those of long-fibre receptors in the medulla (Menzel 1974; Hertel and Maronde 1987). Neurons in the proximal medulla and distal lobula exhibit spectral opponency in their phasic (i.e. ON–OFF antagonism) or tonic responses (Kien and Menzel 1977b; Hertel 1980; Riehle 1981). Other neurons in the medulla are not colour-opponent with both excitatory and inhibitory responses, yet are wavelength-specific, responding only to a very narrow range of wavelengths in UV, blue or green part of the spectrum (narrow-band neurons, Kien and Menzel 1977b; Hertel 1980). Work by Paulk et al. (2009) provided new insights recording from the bumblebee optic lobes. They compared immunohistochemical stainings of branching patterns with intracellularly recorded colour responses in identified neurons in the same layer of the medulla. Similar to the honeybee studies, colour-opponent responses in large-field and amacrine neurons were found in proximal layers of the medulla, where also high levels of GABAergic and serotonergic staining were observed. This indicates that inhibitory and modulatory processes related to coding spectral and temporal aspects of colour processing might take place in the proximal medulla (Paulk et al. 2009).

Most tonic colour-opponent neurons in the honeybee have been found in the proximal lobula and in medulla-extrinsic interneurons with projections to the protocerebrum via the posterior optic commissure (Hertel 1980; Riehle 1981; Hertel et al. 1987). Overall, the response patterns of colour-sensitive lobula neurons seem to be more diverse and complex, including opponencies between single or combined receptor signals and one instance of double spectral opponency with differential input from each eye (Kien and Menzel 1977b; Hertel 1980; Riehle 1981; Hertel et al. 1987; Hertel and Maronde 1987; Yang et al. 2004; bumblebees—Paulk et al. 2008).

The receptive fields of colour-opponent neurons recorded in the proximal medulla were completely homogeneous (Kien and Menzel 1977b; Hertel 1980), except for one large-field neuron that had a dorso-ventrally segregated receptive field with combined spatial and phasic spectral opponency (Hertel 1980). In the lobula colour-sensitive neurons showed variations in responses across locations in the receptive field, but the spatial organisation was not clear-cut. Overall, there are no indications that spatial antagonism is a characteristic feature of colour-sensitive neurons in bees (Kien and Menzel 1977a, b; Hertel 1980; Yang et al. 2004), which is in stark contrast to colour-coding midget ganglion cells in primates, which have a spatial centre-surround organisation of their receptive fields.

The receptive fields of intracellularly recorded colour-sensitive neurons vary in size but tend to be rather large, above 30° (Hertel 1980) or 60–70° and above (Kien and Menzel 1977b). This corresponds to the wide branching patterns of colour-sensitive large-field and amacrine neurons in the medulla (Hertel 1980; bumblebees—Paulk et al. 2009). However, Hertel (1980) mentioned that some narrow-band neurons of the medulla had small receptive fields but did without providing any further specifications. The functional significance of such coarse raster for coding details of objects and visual scenes is still unknown, and it remains unclear how spatial aspects of colour vision might be coded.

Extrinsic tracts from the optic lobes project to the calyces of the mushroom bodies, the optic lobe of the contralateral eye and various areas of the proto- and deutocerebrum (Erber and Menzel 1977; Homberg 1985; Gronenberg 1986; Hertel and Maronde 1987; Hertel et al. 1987; Maronde 1991; Ehmer and Gronenberg 2002). However, colour-sensitive interneurons have so far only been found in the anterior optical commissure (AOC), projecting to the mushroom bodies and the lateral protocerebrum; and in the posterior optical commissure (POC) that joins the medullae of both eyes (Hertel et al. 1987; Menzel and Backhaus 1991, bumblebees—Paulk and Gronenberg 2008). In the mushroom bodies of the honeybee brain, interneurons originating from the medulla innervate mostly the collar region of the calyces, whereas lobula neurons have more numerous projections to the basal ring (Ehmer and Gronenberg 2002). There is partial overlap of projections at the border of the two layers. The functional consequences of this segregated input from the medulla and lobula to the mushroom bodies are unclear. Very recently colour-specific responses were also found in the anterior optical tubercle of the anterior protocerebrum of honeybees, which relates to previous findings from intracellular recordings in locusts and the neuroanatomy of the anterior optical tubercle in bumblebees (Kinoshita et al. 2007; Pfeiffer and Kinoshita 2012; Mota et al. 2013). Whilst colour input to the mushroom body is most likely related to colour learning and object recognition (Menzel 1967; Menzel and Backhaus 1991), colour processing in the anterior optical tubercle is hypothesised to be functionally significant for disambiguating directions in navigational compass mechanisms (Kinoshita et al. 2007).

The neurophysiological data obtained need to be expanded to further investigate the interactions and functions of different types of visual interneurons, before a detailed neural model of colour processing in the bee brain can emerge. Some facts have, nevertheless, been clearly established. There is solid evidence for the existence of colour-opponent, colour-sensitive and broadband interneurons in the periphery of the visual system, which serve as the main neural substrate for the segregated colour and brightness pathways in the bee. Achromatic vision has a higher sensitivity and resolution as compared to the colour vision system (Menzel 1981; Srinivasan and Lehrer 1984, 1988; Giurfa et al. 1996; Hempel de Ibarra et al. 2001), which can be attributed to the preponderance of L-receptors that occurs in each ommatidium across the retina. Colour information is relayed to different areas of the brain which link with its diverse behavioural functions in foraging and navigation behaviours.

## Models of colour discrimination

Daumer (1956) laid the foundations for psychophysical investigations of colour vision in the bee. He pioneered training methods that involved individual bees as opposed to the mass testing commonly used until then. Furthermore, he developed methods to carefully control light stimuli while systematically varying their spectral properties. His work and the first intracellular recordings by Autrum and von Zwehl (1964) strongly suggested that three photoreceptors are involved in the discrimination of colours. Von Helversen (1972) expanded upon this evidence. He tested the fine-scale colour discrimination ability of bees by meticulously measuring their spectral sensitivity and wavelength discrimination functions. This was the first study of wavelength discrimination in a non-primate animal. Adopting similar training methods as Daumer (1956) and Menzel (1967), he trained bees individually to a rewarded horizontal disc and tested them in unrewarded tests. Stimuli were presented in multiple locations to prevent the development of spatial preferences. Initially bees were trained to an unilluminated disc against another disc that was illuminated from the back by monochromatic light. The intensity of the unrewarded disc was reduced until bees confused the stimuli, to match the subjective brightness of lights of different wavelengths for the main wavelength discrimination experiment. Later studies have demonstrated that spectral sensitivity responses are mediated by chromatic mechanisms and cannot be used to adjust the stimuli to equal brightness (Brandt and Vorobyev 1997). Despite this drawback in the method, the function measured by von Helversen (1972) can be considered to represent the wavelength discrimination function because bees were not sensitive to changes in the light intensity in a comparable experiment by Daumer (1956). These spectral sensitivity and wavelength discrimination functions clearly evinced the trichromatic nature of honeybee colour vision. The spectral sensitivity peaks in the UV, blue and green parts of the spectrum correspond to the three types of photoreceptors. Wavelength discrimination was best where the flanks of the photoreceptor sensitivity curves are steepest. The wavelength discrimination function of the honeybee has two minima: one located between the peaks of S- and M-, the other located between the peaks of M- and L-receptors. The smallest wavelength difference that bees discriminated with 70 % accuracy was 4.5 nm, approximately around 400 and 500 nm in the bee.

Later, psychophysical work adopted several conceptual approaches to investigate the coding principles underlying colour discrimination in the honeybee, which led to the formulation of a number of colour vision models. This work also included the demonstration of colour constancy in the honeybee, i.e. the ability to perceive colours as unchanged under various conditions of illumination (Neumeyer 1981; Werner et al. 1988). One of the first proposed models of colour constancy is the von Kries transformation (Vorobyev et al. 2001b). It assumes that signals of photoreceptors are scaled so that the colour of illumination remains invariant. Such an algorithm can be implemented by receptor adaptation and is a very simple mechanism because it does not require sophisticated neural processing. The von Kries model yields predictions that agree with results of behavioural experiments (Neumeyer 1981; Werner et al. 1988), but it cannot achieve perfect colour constancy. Nevertheless, the algorithm works well under naturally occurring changes in illumination, maintaining consistent perceptual colourations of flowers (Vorobyev et al. 2001b).

Daumer (1958) used the three excitation functions that were determined as primaries for colour mixing in his earlier work (Daumer 1956) to represent coloured stimuli in a perceptual colour space for bees based on the rationale of the Maxwell triangle. Each colour corresponds to a specific ratio of these three primaries and can be represented by the coordinate in the three-dimensional space where its vector crosses a two-dimensional unity plane with triangular boundaries (the Maxwell triangle). A similar approach was also implemented by Neumeyer (1980, 1981) who investigated colour contrast and colour constancy in bees (Table 1). She used three spectral sensitivity functions that were derived from psychophysical colour discrimination experiments to define a receptor-based colour space for the bee (e.g. Wyszecki and Stiles 1982; Backhaus 1998). In the Maxwell triangle, stimuli that differ only in their intensity occupy the same locus, whilst stimuli that differ in their chromaticity occupy different loci. Whilst the triangle provides a quantitative description of stimuli for systems with different spectral inputs, it is important to note that Maxwell triangle is not intended to describe perceptual differences between colours (Backhaus 1998).

**Table 1:** Models of colour discrimination for the honeybee

Model	Publications	Basic assumptions
Maxwell triangle	Neumeyer (1981)	No specific colour-coding mechanisms
COC model (colour-opponent coding model)	Backhaus et al. (1987), Backhaus (1991)	Hyperbolic transformation of quantum catches; city-block metric
Hexagon model	Chittka (1992)	Hyperbolic transformation of quantum catches; euclidean metric
GCO model (general colour-opponent coding model)	Brandt and Vorobyev (1997)	Receptor signals are linear functions of quantum catches; riemannian metric
RNL model (receptor noise-limited colour-opponent model)	Vorobyev and Osorio (1998), Vorobyev et al. (2001a)	Receptor signals are given by logarithms of quantum catches; receptor noise limits discrimination: (a) with constant noise-to-signal ratio (receptor noise obeys Weber’s law), or (b) with square root noise-to-signal ratio (receptor noise obeys Rose–de-Vries law)

More models describing colour vision in bees have since been proposed (Table 1). These models differ in their assumptions about the processing mechanisms underlying colour detection and discrimination. All models follow the basic ideas of classical metric theory of human colour discrimination (Helmholtz 1896; Schrödinger 1920), representing quantitative differences through the separation of colour loci in a colour space; the larger the separation, the higher the probability that stimuli are discriminable. Hence the distance between colours can be measured in the terms of just noticeable difference (jnd), which corresponds to a certain probability of discrimination (often 75 %). The models that were derived from behavioural or electrophysiological data (Backhaus 1991; Brandt and Vorobyev 1997; Vorobyev et al. 2001a) represent a substantial improvement over approaches that were based purely on theoretical considerations (Neumeyer 1980; Chittka 1992).

The models of colour discrimination generally do not describe perceptual difference between colours. However, as the distance between loci in this colour space is positively correlated with the discriminability of colours, it is often assumed that the separation between colour loci corresponds to the perceptual difference between colours. This assumption must be taken with caution for the following reasons: (1) the behavioural measure to which the ‘perceptual’ difference corresponds is usually undefined, and (2) the perceptual difference between colours may differ from the colour difference measured in just noticeable differences (jnds).

The models assume that signals from the three receptor types are compared via two independent colour-opponent mechanisms to code the chromatic aspects of a stimulus and ignore detection and discrimination of stimuli on the basis of achromatic mechanisms. While this assumption is valid for many experimental settings, bees are also able to detect and discriminate stimuli on the basis of achromatic cues alone, as will be discussed later (Giurfa et al. 1997; Giurfa and Vorobyev 1998; Niggebrügge and Hempel de Ibarra 2003).

The two models of honeybee colour vision that are based on experimental data—the COC model (Backhaus 1991) and the RNL model (Vorobyev and Brandt 1997; Vorobyev and Osorio 1998; Vorobyev et al. 2001a)—describe behavioural data well. Both models assume that general neural noise sets the limit for discrimination of light stimuli (Backhaus and Menzel 1987; Menzel and Backhaus 1989; Backhaus 1991, 1993; Brandt and Vorobyev 1997; Vorobyev et al. 2001a), and consequently very close loci cannot be discriminated if they fall within the noise-delimited area around any colour locus. However, the models disagree in their more detailed assumptions about the source of this noise. The COC model of Backhaus (1991) postulates that colour discrimination is limited by the neural noise originating in the two colour-opponent mechanisms and regards noise in receptor mechanisms as being negligible. Conversely, the RNL model of Vorobyev et al. (Vorobyev and Brandt 1997; Vorobyev and Osorio 1998) is based on the assumption that colour thresholds are determined by the noise in receptor mechanisms and that the noise originating in colour-opponent mechanisms is negligible.

The parameters of the COC model have been inferred from multidimensional scaling of colour choices in behavioural experiments (Backhaus et al. 1987). Based on the evaluation of the neurophysiological evidence, Backhaus and Menzel (1987) proposed that the neural substrate for the two opponency mechanisms in the honeybee is represented by those neurons in the medulla that respond differentially to UV light versus blue and green (S−/M+L+, S+/M−L−) and to blue versus UV and green light (M−/S+L+, M+/S−L−) (Kien and Menzel 1977b; Hertel 1980). These receptor opponencies resemble the colour-opponent primaries derived by Daumer (1956) in additive colour-mixing experiments. It should be noted, however, that additive colour matching can also be explained by the variations of signals from the three photoreceptors. Next Backhaus et al. (1987) attempted to determine the nature of the colour-opponent mechanisms from the results of multidimensional scaling of the honeybee choices and demonstrated that the derived parameters were in good agreement with the properties of these two types of neurons, recorded by Kien and Menzel (1977b). Nevertheless, it is difficult to deduce the properties of neural mechanisms, such as colour opponency, from psychophysical data both in humans and in non-human animals (see also Vorobyev and Osorio 1998; Kelber et al. 2003).

The parameters of the RNL model are inferred from electrophysiological recordings of noise in photoreceptor cells of the honeybee (Vorobyev et al. 2001a). Contrary to the COC model, the RNL model does not specify the nature of the two colour-opponent coding mechanisms that compare receptor signals.

The first step in the calculation for either model is the quantification of receptor signals as the number of effectively absorbed quanta (so-called quantum catches), *Q*_*i*_ (Eq. 1 , *i* = S, M, L-receptors), based on the photoreceptor spectral sensitivity, illumination and reflectance functions. Quantum catches are then transformed according to basic assumptions of the models (Table 1). The COC model applies a hyperbolic transformation, which implies saturation of receptor signals at certain level. The RNL model instead uses a logarithmic transformation which corresponds to the dynamic range of stimulus intensities used in experiments, particularly with reflective surfaces. The final step is the conversion of the three transformed receptor inputs into a colour locus with coordinates XY in a two-dimensional perceptual colour space, and the calculation of distances between colour loci (ΔS) therein, for which different metrics are employed. Details of calculations are given in the Appendix.

### Limitations of model predictions

Colour vision models make different assumptions about colour-coding processing (for a detailed discussion see Brandt and Vorobyev 1997; Kelber et al. 2003). Whilst predictions across models may be consistent in some cases for a subset of coloured stimuli in a particular task, there are also instances in which models differ substantially in their predictions or fail to correctly predict detection or discrimination performance.

Brandt and Vorobyev (1997) applied a metric analysis to the behavioural spectral sensitivity curve recorded by von Helversen (1972) for individual bees. They tested a variety of models presenting specific hypotheses about the processes underlying colour discrimination at receptor and/or post-receptor stages, with and without interactions between receptor signals, and compared their predictions with the behavioural data, taking into account its variance. The analysis confirmed that colour discrimination in bees does not require more than two post-receptor mechanisms and found that several models incorporating post-receptor interactions, or colour opponency, accurately predict the bee’s spectral sensitivity curve. Amongst these is the COC model by Backhaus (1991). Unlike other models that commonly use Euclidian metrics to determine distances between colour loci in a colour space, this model is special in that it applies a city-block metric (given the specificity of its assumption about the types of colour-opponent mechanisms), e.g. distances along the two space axes are added up. Whilst this model predicted the spectral sensitivity curve with sufficient accuracy, it was not the case for the hexagon model (Chittka 1992), notwithstanding that it produced predictions that roughly fitted discrimination data obtained in colour discrimination tests at the hive entrance for several bee species (Chittka et al. 1992).

The metric analysis further evinced that achromatic channels are either not involved at all or the sensitivity of the achromatic channel is very low (Brandt and Vorobyev 1997), confirming the conclusions reached by Daumer (1956), Menzel (1967), von Helversen (1972) and Backhaus et al. (1987). Thus, an important feature of honeybee colour vision is the profound separation between chromatic and achromatic mechanisms. When bees are tested with stimuli subtending large visual angles or are allowed to approach the stimulus, they exclusively use chromatic vision, but smaller stimuli are detected and discriminated by an achromatic mechanism that is mediated by the L-receptor alone (Giurfa et al. 1996, 1997; Giurfa and Vorobyev 1998). Interestingly, the low sensitivity of achromatic vision for stationary stimuli subtending large visual angles is also a feature of visual systems of many diurnal animals, such as primates (including human beings) and at least some species of birds (Vorobyev and Osorio 1998). Therefore colour discrimination in many animals can be modelled using the RNL model (Vorobyev and Osorio 1998) that was originally proposed to describe colour discrimination in the honeybee (Vorobyev and Brandt 1997).

An interesting case where discrepancies in predictions of different models arise is the discrimination of dim and bright colours (Vorobyev et al. 1999; Hempel de Ibarra et al. 2000). Bees were trained to choose a large green or blue target against a UV-grey or UV-white background (and vice versa). For combinations with strong chromatic contrast, their performance was better than one would expect assuming that signal-to-noise ratios improve at higher light intensities. The RNL model predicted the results best, because it uses a logarithmic transformation of receptor signals. In contrast, models implementing a hyperbolic transformation of receptor signals (COC, Hexagon) predicted an impairment of performance for highly contrasting colours, which was not confirmed in detection experiments (Hempel de Ibarra et al. 2000). Other Y-maze experiments, where the detectability of coloured and achromatic discs against dim or bright grey backgrounds was compared, provided additional evidence that the improved performance was not achieved through the involvement of the L-receptor mechanism (Niggebrügge and Hempel de Ibarra 2003). Models make accurate predictions if they correctly represent the principles of biological processes and if they are operated within the limits of their assumptions. Preferably models should be based on experimental data, such as the RNL and the COC models. Using unsuitable models or using models wrongly can lead to significant errors and unreliable speculations (for an example and discussion see Kevan et al. 1996; Waser and Chittka 1998; Chittka 1999; Vorobyev 1999; Vorobyev et al. 1999).

### Colour thresholds: models and experiments

How do distances in a colour space relate to the bee’s ability to discriminate between colours? As presented above, colour spaces are mathematical formulations of models that make specific assumptions about the nature of the coding processes underlying colour discrimination. A key distinction lies in the determination of the stage that limits colour discrimination performance (Fig. 1). Ideally, in a colour vision system, even when operating under photopic conditions in which photon and receptor noise is less limiting than in dim light conditions, noise introduced at the post-receptor stage should not exceed that of the photoreceptors (Vorobyev and Osorio 1998).

Photoreceptors are metabolically costly, and it can be assumed that their noise levels are subject to strong selection (Laughlin et al. 1998; Laughlin 2001). Colour-coding neurons can avoid impairment of discrimination through signal amplification with high gain (Brandt and Vorobyev 1997; Vorobyev and Osorio 1998; Vorobyev et al. 2001a). Indeed, recordings from bee monopolar cells in the lamina show that their gain is higher than that of photoreceptors (Menzel 1974; de Souza et al. 1992). The temporal resolution of colour vision, determined to be at 100 Hz in free-flying bees through measurements of the flicker-fusion frequency (Srinivasan and Lehrer 1985), is close to the limit imposed by the temporal resolution of single photoreceptors (Autrum and Stöcker 1950; Srinivasan and Lehrer 1984). This indicates that colour discrimination is not compromised further in this fast-flying insect, and temporal factors are unlikely to play a major role in limiting colour discrimination.

Backhaus and Menzel (1987) used receptor noise estimates to explore the idea formulated by Helmholtz (1896) that the input stage (photoreceptors) limits colour discrimination. However, direct measurements from honeybee receptors revealed much higher noise ratios than assumed by Backhaus and Menzel (Vorobyev et al. 2001a). The RNL model postulates the receptors as the main source of noise in a colour-opponent coding system. The model has no free parameters, as the level of noise in receptor mechanisms has been estimated from physiology (Vorobyev et al. 2001a). However actual thresholds may vary, and variations between animals and within an animal tested on different days have been observed in many studies (e.g. von Helversen 1972; Vorobyev et al. 2001a). Such variations can be explained by the summation of signals of individual photoreceptors that improve signal-to-noise ratios. How many receptor signals might be summed up maximally can be determined behaviourally in detection experiments by measuring the limits of spatial resolution of the bee’s colour vision system (Giurfa et al. 1996). The comparison of different levels of summation showed that the behaviourally determined upper limit is never exceeded (Vorobyev et al. 2001a). Another important factor determining the variability of thresholds is the change of decision rule adopted by an animal, which depends on the motivation, the training conditions, the cues presented in the tests, and on the cost and benefits of correct detection (discrimination) versus the error in the detection. These considerations have been formally considered within the RNL model (Vorobyev et al. 2001a).

The RNL model is based on the assumption that visual information is not lost in the brain, i.e. the brain processes the information ideally. “Ideal observer” models of this kind are based on signal detection theory, which relates the probability of detection of the signal to the false alarm rate. The ideal observer models postulate that the honeybee makes a decision based on the perceived difference to the memorised rewarded stimulus exceeding a certain response criterion. When two stimuli are presented, the honeybee decides which one is more likely to be rewarded. If neither stimulus achieves the response criterion, the stimuli are chosen with equal probability. Due to noise, the perceived distance between the stimuli may exceed the response criterion even if the stimuli are identical. This leads to a false alarm. To decrease the probability of false alarms the response criterion must be increased, which leads to the increase of threshold. Therefore the actual threshold reflects the trade-off between the requirement to increase the probability of detection and decrease the probability of false alarm. Formal consideration of these factors leads to equations that relate the probability of detecting a stimulus to the distance of the stimulus from background for any given value of the false alarm (Vorobyev et al. 2001a).

A major problem for testing colour discrimination thresholds is excluding the use of other sensory cues, which is particularly challenging when using broadband reflection stimuli such as coloured or printed paper. In the case of monochromatic stimuli, such factors are easy to control. Therefore one must be cautious when prolonged training is used for stimuli that are predicted to be similar because bees may pick up additional cues, particularly when ‘incorrect’ choices are punished with quinine thus forcing the bees to use any other information that helps to avoid the aversive reward (e.g. Dyer and Neumeyer 2005; Avarguès-Weber et al. 2010; Wang et al. 2013). This is particularly critical, if stimuli are not well-matched for achromatic L-receptor contrast in such experiments, because bees could become unusually sensitive to achromatic contrast for solving the discrimination task, which none of the colour vision models would be able to predict.

To investigate colour vision it is generally beneficial to use narrow-band colour stimuli, produced with monochromatic filters or light sources, to carefully control stimulus quality and stimulate across the whole visible range, including the UV. While broadband-reflecting stimuli are easier to obtain with printed papers or coloured materials, it is important to understand that reflecting stimuli and illumination functions cannot be measured to the degree of accuracy that can be obtained using monochromatic stimuli. Furthermore, the spectra of many reflecting materials depend on the viewing angle, and this parameter is difficult to control. Finally, the conclusions derived from experiments with reflecting stimuli critically depend on accuracy of the estimate of the spectral sensitivity of the honeybee. Therefore, experiments have to be designed carefully and caution is needed in interpreting the results of behavioural experiments obtained with reflecting stimuli, especially when the stimuli are designed to occupy close loci in the colour space.

## Behavioural functions of colour vision in bees

### Colour learning

To make efficient foraging decisions bees associate visual, olfactory and tactile features of floral displays with varying qualities of reward. Until Menzel (1967, 1968, 1969, 1979b) started to systematically investigate colour learning, the mechanisms of learning and memory processes involving colour vision were not the focus of bee vision research, although many studies trained bees with colours (reviewed by von Frisch 1965) or used colours to study learning in the context of spatial orientation (Opfinger 1931). Using monochromatic lights to control the colours of targets and training bees individually, Menzel (1967, 1968, 1969) demonstrated that bees learn colours extremely quickly. After a single colour–sucrose pairing, in the case of blue-violet colours (e.g. 413, 428 nm), bees already responded very strongly to the training colour in unrewarded tests, forming memories that could last up to 6 days. Other colours, particularly blue-green (e.g. 494 nm) required very few pairings before they were thoroughly discriminated and long-term memories were formed. Bees could learn new colours after initial training, and also performed successfully in colour-reversal tasks requiring simultaneous memories of two colours. Later work revealed further details about the formation of short- and long-term memories in honeybees and the role of reward timing and duration (Menzel and Erber 1972; Erber 1975; Menzel 1979b). These experiments evinced a surprising flexibility in colour learning that was unexpected, as insects were traditionally considered to possess no or very limited learning abilities.

Laboratory studies with naïve forager honeybees revealed a spontaneous preference for targets reflecting in the short-wavelength range around 410 nm (Giurfa et al. 1995), but colour learning processes very quickly override these tendencies and dominate colour choice. Bees learn every colour well, including UV-reflecting white and grey (Daumer 1956; Menzel 1967; Hempel de Ibarra et al. 2000), but colour choices can become rather complex if extended training regimes are applied that expose a bee to variations of rewarded and unrewarded or even punished colour signals. Whilst such experiments may demonstrate the abilities of bees to cope with complex training conditions, they do not lead to data sets that would be suitable for modelling approaches based on psychophysical rules, as discussed above.

In simple training experiments, bees are able to generalise and categorise colours, which is likely to optimise their foraging behaviour dealing with natural variation in colours of individual flowers (Menzel 1967, 1969, 1985; Backhaus et al. 1987; Giurfa 1991; Greggers and Mauelshagen 1997). The level of generalisation depends on the learning conditions. After differential conditioning, when bees have to distinguish between two colours, their generalisation curve will be narrower, as compared to bees trained with only one colour (Giurfa 2004), confirming what is generally known from many other associative learning studies. Colour generalisation in honeybees can also be affected by a peak shift towards a novel colour, as shown by Martínez-Harms et al. (2014). In this work the RNL model colour space was used to characterise a perceptual continuum among three colour stimuli—a training colour, an unrewarded alternative colour and a novel colour that was similar to (but discriminable from) the training colour. If the novel colour was further away from the unrewarded alternative than the training colour, bees in the test would prefer the novel colour over the learnt one. Bees tested in a control condition showed that a different position in the colour space did not produce this effect, as predicted by the peak shift phenomenon. Peak shift is found in human perception and in the visual perception of many animals; however, it has been little studied in insects. It is particularly interesting in the context of pollination, where it could have a significant impact on the evolution of flower colours by influencing pollinator-induced selection for discriminable colours (Lynn et al. 2005; Martínez-Harms et al. 2014).

The associative learning protocol of proboscis extension response (PER) conditioning with visual stimuli in harnessed honeybees (e.g. Kuwabara 1957; Masuhr and Menzel 1972) has recently been successfully revisited in the context of colour learning. Interestingly, under such conditions colour discrimination and generalisation responses appear to be less elaborate than in free-flying bees (Hori et al. 2006; Niggebrügge et al. 2009; Jernigan et al. 2014), which further indicates that the behavioural context is important for learning and memorising colours.

### Detection of coloured objects

One of the major functions of the bee’s visual system is the detection of flowers, which vary in size and differ in colour and brightness from their background. For a long time, it was generally assumed that to be more conspicuous, flowers should increase the size of their display and its contrast with the background, but these ideas remained largely untested. Giurfa et al. (1996) set out to determine how spectral features increase target detectability by performing a series of detection experiments in a Y-maze with a dual-choice task. Honeybees had to detect a rewarded coloured disc of various sizes presented on a grey vertical back wall in one of the two arms, whilst the other arm contained just the grey wall. As long as the stimulus was large enough to be seen by the bee from a distance, the bee was able to select the correct arm. By increasing the distance, the angular size of the disc was successively decreased until the bee’s choices fell to chance level, indicating that the disc became invisible. The advantage of using a Y-maze is that the angular size of targets can be controlled. Thus, coloured discs became visible to bees only when they made a choice, and not before while approaching from further away, as in open displays (e.g. Lehrer and Bischof 1995).

Colours were selected to differ spectrally, such that receptor-specific (S-, M- or L-) contrasts were individually matched to the background. It was expected that the detectability measured as angular detection limits would vary as a function of contrast strength and thus any particular receptor critically involved in colour detection would be identified. However, an unexpectedly simple pattern emerged from the responses of the bees. Discs with colours that did not provide L-receptor contrast were detected over a shorter distance range than those with L-receptor contrast, and two corresponding angular detection thresholds of 15° and 5° were determined. Similarly, Lehrer and Bischof (1995) determined a detection threshold of 5° for coloured discs with L-receptor contrast using an open arena. The results showed that an L-receptor-mediated mechanism enhanced the detectability of the coloured disc, whereas disc diameter, the strength of chromatic contrast and the strength of any receptor-specific contrast (or sum of receptor signals as a possible measure for overall intensity) could not account for the results. Discrimination experiments showed that detection and discrimination over a short distance range, i.e. when coloured discs subtend large visual angles, were mediated by chromatic cues, whilst further away, at small angular extents, achromatic L-receptor contrast was required for detection and discrimination (Giurfa et al. 1997). Similar detection thresholds were obtained when replicating the experiments with honeybees in various subsequent studies (Hempel de Ibarra et al. 2001; Dyer et al. 2008; Wertlen et al. 2008).

**Fig. 3:**
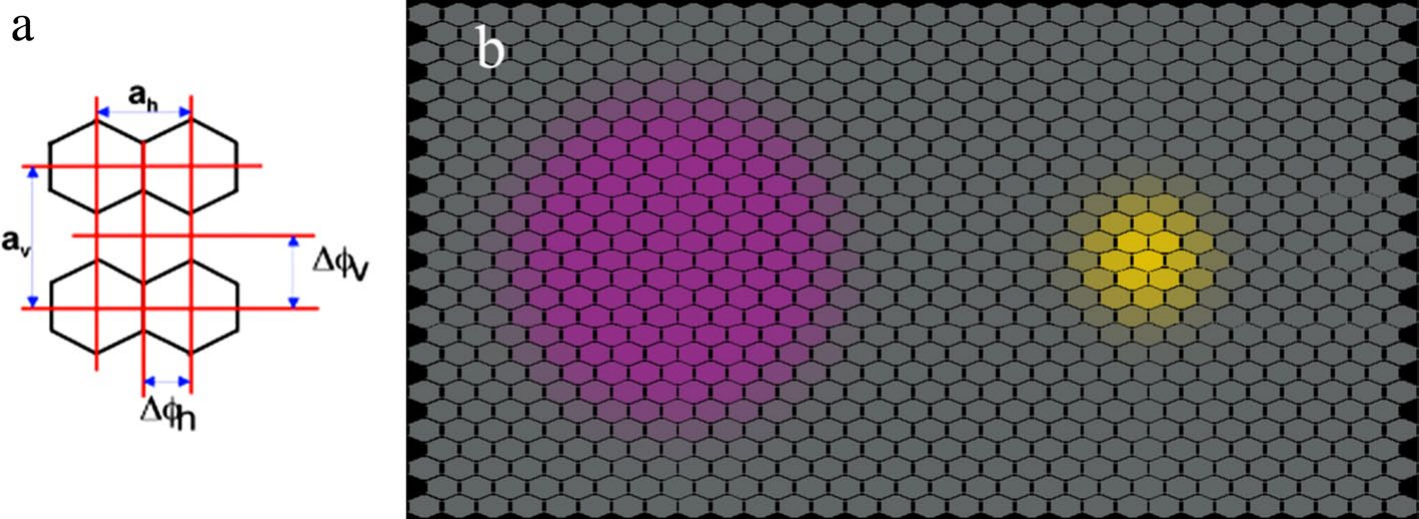
Projection of two circular stimuli onto the ommatidial lattice of the frontal region of the honeybee compound eye. **a** Facet lens pattern: *a*_h_ and *a*_v_ are the primitive translation vectors in the horizontal and vertical direction, respectively. Δ*Φ*_h_ and Δ*Φ*_v_ are the interommatidial angles in the horizontal and vertical directions, respectively. **b** Stimuli at the achromatic and chromatic detection thresholds which is at 5° (*yellow*) and 15° (*violet*) of visual angle, respectively. Relative excitations of ommatidia with respect to that of the ommatidium projecting onto the centre of the stimuli are shown: the stronger the colouration the higher the excitation of the ommatidia. Reproduced from Vorobyev and Hempel de Ibarra (2012), with permission from Springer

To estimate how well this performance matched the best possible optical resolution, Giurfa et al. (1996) calculated how many ommatidia were imaging the disc at the detection threshold using an optical model of the honeybee ommatidial lattice (Fig. 3). The lens apparatus of the ommatidia is fixed, so the number of ommatidia looking at a target varies with the viewing distance. The optical lattice of the ommatidia in the frontal part of the bee eye, where it has its highest optical resolution (Seidl 1982), is described by the interommatidial angles (horizontal Δ*Φ*_H_ = 0.9° and vertical Δ*Φ*_V_ = 1.6°, Kirschfeld 1973), and by the acceptance angle measured with intracellular recordings from the honeybee photoreceptors (Δ*ρ* = 2.6°, Laughlin and Horridge 1971). The calculations showed that more than 59 neighbouring ommatidia had to be excited to render the disc detectable for the chromatic visual system, whereas 7 ommatidia were sufficient to detect the disc if it had L-contrast. This is puzzling given that the optical structure of the insect compound eye could optimise chromatic and achromatic detection by matching object detection limits to the acceptance angle of a single ommatidium. A possible explanation is that bees sacrifice spatial resolution to improve contrast sensitivity. Indeed, a direct comparison of behavioural colour thresholds with the electrophysiological measurements of receptor noise indicates the improvement of the signal-to-noise ratio by summation of signals of photoreceptor cells (Brandt and Vorobyev 1997; Vorobyev et al. 2001a), providing an explanation for the much lower spatial resolution of the chromatic detection mechanism. It is likely that every ommatidium of the heterogeneous honeybee retina within the large receptive field of the chromatic detection units is involved in the summation of chromatic signals, though this has yet to be demonstrated.

The question then arises: Why is achromatic detection not optimised to achieve the highest possible resolution as determined by the optical structure of a single ommatidium? A possible answer was provided by another set of detection experiments employing an achromatic stimulus (pink to a human observer) on a grey background that only presented L-contrast. Giurfa and Vorobyev (1998) found that bees were able to detect the disc only when it subtended between 5° and 15°, with a maximum in the performance curve around 7°. The disc was not seen by the bees when it was close to them, subtending visual angles above 15°. The fact that the angular sensitivity of the achromatic detection mechanism has a higher and lower threshold indicates that receptor signals from different ommatidia are not simply summed up within a receptive field, but interact. A linear model of detector units with Gaussian centre-surround receptive fields predicts the detection performance of the bees well (Giurfa and Vorobyev 1998). Such detectors are sensitive to borders and small objects, and visual neurons with centre-surround receptive fields seem to be common in the periphery of visual systems (Laughlin 1987; Hubel 1988). In the honeybee they could be located in the lamina. Neighbouring cartridges are interconnected via laterally spreading laminar interneurons (Ribi 1976), which are prime candidates for forming centre-surround receptive fields. The heterogeneity of the honeybee retina does not affect such considerations, because every ommatidium contains six L-receptors (Wakakuwa et al. 2005).

A linear increase of detectability with increasing signal strength, as predicted by the centre-surround detector model, however, was not observed. All coloured discs had the same detection limit despite large variations in L-contrast (Giurfa et al. 1996; Giurfa and Vorobyev 1998). This indicates that there are non-linearities in the processing of achromatic signals, the nature of which remains unknown. Furthermore, detectability of single discs improves when they are grouped, even with inter-disc distances sufficiently large to prevent optical merging at small angular subtenses (Wertlen et al. 2008). These results indicate that detector units might interact, enabling a varied response to extended distributions of objects across the visual scene. Recent results also suggest that motion parallax may facilitate detection of three-dimensional objects placed at some distance in front of a patterned background (Dittmar et al. 2010; Kapustjansky et al. 2010). These mechanisms seem to be crucial for the bee’s ability to identify single flowers in the natural environment, but more experiments are required to fully understand how object detection mechanisms operate in complex visual scenes.

The tuning of detector units in the achromatic visual system to targets of small sizes also affects the detection and discrimination of coloured concentric patterns when seen from a greater distance (Hempel de Ibarra et al. 2001, 2002). Detectability of coloured patterns composed of a central disc of one colour and a surrounding ring of another was tested similarly to the aforementioned studies using single-coloured discs. The two colours either presented the same L-contrast to the background, thus providing only chromatic pattern cues, or differed in L-contrast. The detection range of chromatic patterns was not affected and resembled that of single-coloured discs.

**Fig. 4:**
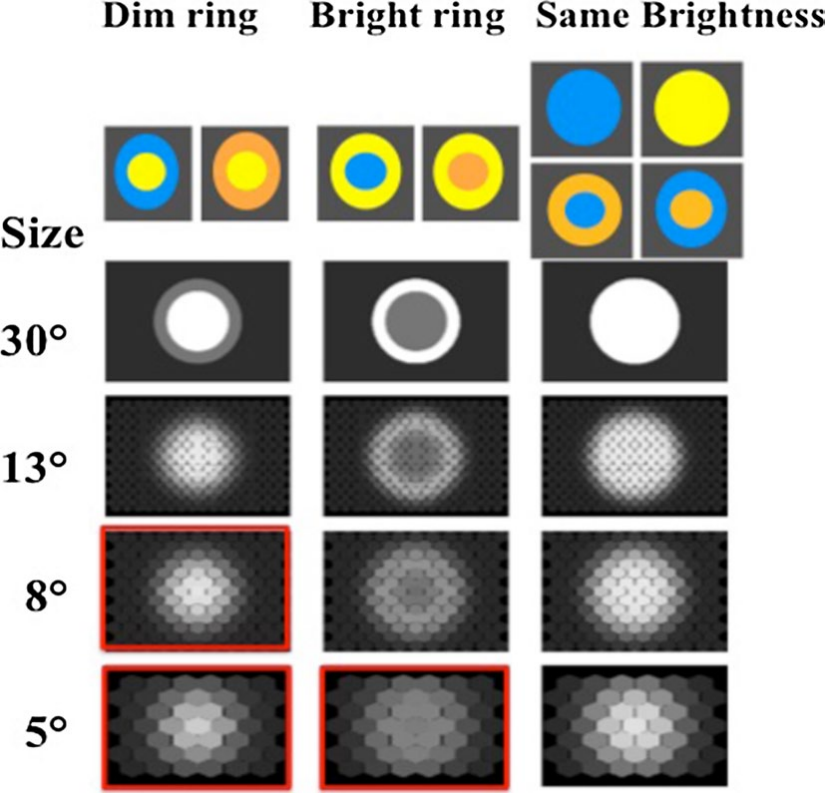
Detection of coloured patterns by honeybees. Patterns composed of two colours which had the same L-receptor contrast (*right column*) were detectable until 5° of visual angle, whereas patterns in which L-contrast was varied were detected over shorter distances. *Red frames* depict angular sizes at which patterns were not detected although equal numbers of ommatidia were excited above threshold. After Hempel de Ibarra et al. (2001)

However, when colours differed in L-contrast, the spatial distribution of colours determined the detection limits of patterns. If pattern colours were arranged such that the central disc was dimmer (had a weaker L-receptor contrast than the ring colour) and was surrounded by a brighter ring, the pattern yielded a detection limit of 6.5°, which was significantly less than for single-coloured discs. The threshold obtained for patterns with the opposite arrangement, e.g. a brighter centre surrounded by a dimmer ring, was even worse, limiting detection to 10°. Figure 4 shows how the patterns appeared to the bee eye when viewed at different distances.

These results provide further evidence that achromatic target detection does not rely on simple signal summation. Neither was detection mediated exclusively by the high-contrast edge of the patterns alone, because in this case the pattern with the high-contrast ring surrounding the dimmer centre should have been detected equally well as the single-coloured discs. It was specifically the spatial distribution of L-receptor contrast edges within the patterns that affected their detectability, and sharper outer borders rendered patterns more detectable than smoothed ones.

When testing the discrimination of these patterns against either single-coloured discs of the same size or their individual elements (ring and small disc) close to the detection limit, it emerged that bees could resolve and recognise the shape of the high-contrasting pattern elements (Hempel de Ibarra et al. 2002). One possible conclusion is that bees saw only the high-contrasting pattern element when targets subtended visual angles close to the detection limit. If this were the case, it could possibly explain the impaired detectability of the pattern with the high-contrasting ring as compared to a single-coloured disc of the same size, assuming that the inner low-contrasting edge of the ring would have an inhibitory effect. However, it cannot explain why the pattern with the high-contrasting central disc was the worst detected. In this case, its detection limit should have been much better, because the size of the central disc was still sufficiently large to facilitate reliable detection at and below 10°, the detection limit obtained for this pattern.

Sharp outer borders render patterns more detectable than smoothed ones, such as in the pattern with the dimmer ring surrounding the bright central disc in our experiments. It appears that pattern discrimination and detection are affected by non-linearities in the processing of L-receptor-mediated visual information, but consistent with the centre-surround detector model (Giurfa and Vorobyev 1998); the results show that edges are critical for detection and discrimination of coloured objects at larger distances.

### Identification of coloured objects

The apparent strong segregation in processing of colour and brightness in honeybee vision might strongly impact on the recognition of coloured, patterned or textured objects when seen from different distances. Major questions that arise from the detection and discrimination experiments are whether bees are ‘brightness-blind’ when viewing large objects at close proximity, and how it can be understood that bees perform very well in traditional learning experiments with large black-and-white patterns and shapes (for a review see Lehrer 1987).

To explore the L-contrast sensitivity in target detection over short distances, Niggebrügge and Hempel de Ibarra (2003) tested bees with a variety of coloured discs subtending 30° of visual angle presented on two different grey backgrounds. Stimuli were selected such that their L-receptor signal contrasted with the darker grey background, but matched the light grey background. The performance improved with increased chromatic contrast (colour distance to the background in the RNL colour space), but was not further increased by the presence of L-contrast. Bees could detect achromatic discs with very strong L-contrast (UV-white and grey on dark backgrounds), yet the performance was very poor compared to most other stimulus conditions. Both results confirm that detection was dominated by the chromatic visual system, and also support earlier findings demonstrating that the achromatic system was rather insensitive at this angular subtense. It explains why an achromatic disc would not be detectable at close distances (Giurfa and Vorobyev 1998), unless it contrasts very strongly (Hempel de Ibarra et al. 2000; Niggebrügge and Hempel de Ibarra 2003), which is in line with the predictions of the linear centre-surround detector model (Giurfa and Vorobyev 1998). As such detector units are sensitive to borders, they are not effective with large uniform stimuli. However, a high-contrast signal in large stimuli would elicit some response. Further evidence for low L-contrast sensitivity at close range is provided by metric analysis of the bee’s spectral sensitivity function (Brandt and Vorobyev 1997) which demonstrates that while a hypothetical achromatic channel in bee colour vision cannot be fully ruled out, its sensitivity is very low and therefore largely ineffective when measuring colour discrimination at this range.

It has been frequently shown that pattern vision in bees is ‘colour-blind’ and relies on the L-receptor-mediated visual system (e.g. Srinivasan and Lehrer 1988; Giurfa et al. 1995; Giger and Srinivasan 1996; Hempel de Ibarra and Giurfa 2003; Lehrer and Campan 2005). This is underpinned by the fact that the L-receptor system is involved in edge detection (e.g. Lehrer 1987) and has a higher acuity (Srinivasan and Lehrer 1988; Giurfa et al. 1996; Hempel de Ibarra et al. 2001). Evidence for processing of pattern information by the chromatic system in the bee is rare, mainly because most studies investigating pattern vision in honeybees used black–white stimuli as targets (for reviews see Wehner 1981; Srinivasan 1994). Only a few explored how honeybees discriminate coloured patterns. Daumer (1958) presented natural flower petals covered by UV-transmitting glass to bees, to investigate whether they could discriminate various colour combinations. Menzel and Lieke (1983) showed that bees discriminate various orientations of the contrast line in patterns that were composed of two half-circles. They found several colour-dependent asymmetric effects in the discrimination performance when the pattern was rotated. This work was complemented by findings from Lehrer (1999) who demonstrated that discrimination performance is improved if bees have to discriminate colours in the lower half of such patterns. These studies show that pattern discrimination is influenced by colour, but to understand the involvement of chromatic mechanisms, the colours featured in the patterns should be matched for L-contrast. In addition, it is important to control viewing conditions. Testing bees in a Y-maze, Hempel de Ibarra et al. (2002) explored whether they could discriminate concentric patterns based on chromatic cues alone. Both colours in these patterns had the same L-contrast, whereas in the control group pattern colours presented two different L-contrasts. After learning the pattern, bees were exposed to the rewarded pattern in one maze arm and an unrewarded alternative in the other arm. The latter were either single-coloured discs of the two pattern colours, the reciprocal pattern, or a checkerboard pattern that provided an optical mixture of the pattern colours. When a pattern subtended visual angles above and close to the chromatic detection threshold, bees discriminated the trained pattern from nearly all alternatives. The results clearly indicated that pattern elements were well resolved, independently of whether or not the pattern contained L-contrast edges. Patterns in which colours were matched for L-contrast were not discriminated below the chromatic detection threshold, which was another important control demonstrating the accuracy of L-receptor matching of the colours (Hempel de Ibarra et al. 2001, 2002). The results strongly suggest that the chromatic system of the bee can be involved in pattern recognition processes, despite its much lower spatial resolution.

It remains an open question how pattern features might be processed neurally in the chromatic pathway. The heterogeneity of the honeybee retina could affect spatial mechanisms in the colour vision system of the bee, but this remains to be established. Receptive field sizes of colour-coding neurons far exceed the behaviourally determined spatial resolution of the chromatic system for flower-like objects and patterns. It has been suggested that response variations across receptive fields measured in colour-opponent neurons could account for spatial antagonisms, or that the variation of spectral opponencies and temporal properties (particularly of the lobula cells) could indicate the need to process spatially complex colour information in colour-coding networks (Kien and Menzel 1977a, b; Hertel 1980; Hertel and Maronde 1987; Paulk et al. 2009).

### Navigation and spatial orientation with coloured landmarks

Whilst bees, as other animals, quickly learn the colours of targets with which they interact, colour may be less important for navigation and spatial orientation. Motion and edge cues seem to provide sufficient information in this context, for which high acuity and fast processing through the achromatic visual system are convenient. For example, Collett and Kelber (1988) used blue and yellow local landmarks to form arrays around an invisible feeder placed in two separate locations. Although learning these colours would have been an easy way to locate the reward, bees mostly relied on panoramic or route cues and ignored landmark colours. In contrast, when honeybees are trained to a single location, they seem to rely more on the colour of landmarks to pinpoint an inconspicuous feeder (Opfinger 1931; Gould 1988; Lehrer 1993). In these studies it cannot be ruled out that bees could have use brightness differences rather than colour to recognise landmarks. However, Cheng et al. (1986) showed that bees have the ability to recognise the colour of landmarks. When trained with a two-coloured array of landmarks, bees in unrewarded tests were guided in their searches by landmark colour independently of variations in brightness. Given their remarkable colour vision, it seems implausible that bees would not utilise chromatic aspects of scenes. However, colour might not always be the best cue, and colours could play a different role for the identification of locations and routes as compared to floral food sources.

## Bee vision and colourful flower displays

Ever since the seminal observations made by Sprengel (1793), Darwin (1862) and Müller (1873), it has been commonly accepted that flowering plants evolved patterns and colourful ornamentation as advertisements to bees and other pollinators. Yet many questions about the communicative relationship between plants and pollinators still remain. More evidence is needed to understand the mechanistic basis of the modulatory effects that floral visual cues have on the foraging behaviour of pollinators, and the evolutionary consequences of selective pressures arising from the animals’ sensory, motor and learning abilities. The study of honeybee colour vision and learning has contributed significantly to the identification of major principles underpinning this fascinating system of biocommunication.

It has often been queried whether honeybee colour vision is representative of hymenopteran pollinators more generally. The best evidence on this question comes from measurements of photoreceptor sensitivities in more than 40 species of bees and wasps using intracellular recordings. This body of work clearly demonstrates that hymenopterans share very similar wavelength sensitivity peaks, which is indicative of a highly conserved pattern at the input stage of the colour vision system (Menzel et al. 1986, 1988; Hertel and Fix Ventura 1985; Peitsch et al. 1992; for reviews see Vorobyev and Menzel 1999; Briscoe and Chittka 2001), thus validating the extrapolation of conclusions based on the honeybee visual system to other hymenopteran pollinators. Nevertheless, it remains of interest to establish whether post-receptor processing might have diverged in significant ways, given that bee species differ in their degrees of generalist versus specialist foraging, live in diverse environments, and are active at different times of day and night. Comparative studies have attempted to systematically evaluate variations in sensory and learning processes between bee species (e.g. Menzel 1985; Chittka et al. 1992; Greiner et al. 2004; Dyer et al. 2008; Wertlen et al. 2008; Somanathan et al. 2008, 2009; Spaethe et al. 2014), and this topic is far from being concluded.

To understand how flowers look to bees, spectral reflectances of petals can be analysed. Early attempts were undertaken by Richtmyer (1923) and Lutz (1924) who were particularly interested in UV reflection, as it is invisible to the human eye. Daumer (1958) developed another method for measuring flower spectra, which was based on spectrometry with monochromatic filters that covered the whole visible range of bees. Technological advances gave rise to the first portable spectrometers, initially purpose-built, and more recently industrially manufactured, for the fast and accurate measurement of flower colours in the lab and the field (e.g. Menzel and Shmida 1993; Chittka et al. 1994).

The analysis of more than 1000 spectra measured with a high-resolution photospectrometer revealed that flowers of modern angiosperms do not use the full gamut of colours that can be seen and discriminated by bees (Vorobyev and Menzel 1999). This is largely due to the limited variation in flower spectra, which have rather smooth broadband shapes with no more than three variations across the bee-visible wavelength spectrum, such as wide peaks, rising or cut-off flanks in the spectral curves. This is rather counterintuitive, because plants should be interested in optimising pollen transfer by being distinguishable from each other and by diversifying their colours as much as possible within the limits of the pollinator’s capacity to discriminate colours. On the other hand, considering that the bee’s colour space is large, competing plants may still be able to diversify sufficiently within parts of it. While bees are able to accurately discriminate flower colours (Chittka et al. 1993; Vorobyev and Menzel 1999), their excellent colour vision is not limited to these stimuli. Due to the equidistant distribution of photoreceptor sensitivity peaks across a wide spectral range, and the steepness and moderate overlap of their spectral sensitivity functions, bees possess a general-purpose colour vision system that is not optimised for discrimination of colours in any particular part of the spectrum. The conclusion to be drawn from these observations is that there is no evidence for a close co-evolutionary relationship between the spectral sensitivity functions of bees and flower colours (Vorobyev and Menzel 1999). It is more likely that flower colours simply diversified to a sufficiently large degree to ensure that pollinators with generic colour vision could operate effectively as pollen vectors.

As bees can easily recognise and distinguish flowers with diverse colours, pollinator-mediated selection resulting in divergence of flower colours appears to be opposed by other factors. These may include the prevalence of generalist pollinators that differ in their behaviour, or differences in visual abilities between the main classes of insect pollinators, such as flies, beetles and butterflies. Additionally, the diversification of pigments and structural elements that determine the colouration of petals could be significantly constrained by biochemical and developmental processes in plants (Rausher 2008). As a consequence, flower colours could be unevenly distributed, falling into few perceptually relevant colour categories for bees. Vorobyev and Menzel (1999) approached this question by analysing whether floral spectra can be clustered based on the shape of their reflectance spectrum. The analysis showed that only three categories appeared to reliably reflect qualitative differences: (1) reflectance spectra with sharp flanks that are white, yellow, orange or red to the human eye, (2) spectra with broad peaks appearing blue and violet to humans, and (3) spectra that increase gradually across the whole or large parts of the spectrum. This approach differs from earlier work analysing these spectra (e.g. Chittka et al. 1994) in that it does not require arbitrary divisions of the wavelength range or a colour space. Overall, the quantitative analysis of flower colour spectra has so far convincingly shown that plants evolved colours as an adaptation for pollination by insects equipped with a generic colour vision system, such as bees, whilst being constrained to maximally diversify reflectance spectra that could result in distinct colours for pollinators.

The diversity of flower colours can be related to the colours of their habitats. For instance, backgrounds composed of foliage, sand or stone vary considerably in their spectral characteristics, which may influence the selection of flower colours in a population (Menzel and Shmida 1993; Menzel et al. 1997). Analysis of the distribution of L-receptor signals of flowers collected from dissimilar habitats did not reveal any difference in their L-receptor signals (Menzel et al. 1997). This can be explained by the finding that the minimal angle from which bees can detect targets does not depend on the magnitude of L-contrast, once it exceeds a threshold value (<30 % in Giurfa et al. (1996), for a review see Giurfa and Vorobyev 1997).

Another intriguing question is whether plants that flower together always differ in colour. Often this is the case. For example, in several European habitats the majority of co-flowering plants displayed a range of colours that was easily distinguishable for bees (Gumbert et al. 1999) allowing them to potentially recruit flower-constant pollinators and prevent inappropriate pollen transfers from other plants. Sometimes however, flowers are indistinguishable in colour for bees and benefit from sharing a colour. The rewardless orchid, *Orchis boryi*, has similar colouration to the nearby prevailing flowers, and thus achieves to receive sufficient visits by bees to reproduce successfully (Gumbert and Kunze 2001). This case of generalist Batesian mimicry—where several plant species serve as models for the deceiving mimic—is one possible strategy that flowers have evolved to take advantage of shared colour displays. Another variation of floral mimicry is found when the mimic is associated with a single model or a group of closely related model species, such as in the case of *Turnera sidoides pinnatifida* that grows in similar habitats as various mallow species. In separate populations *T. s. pinnatifida* has different colours which resemble the colours of the prevailing malves (Benitez-Vieyra et al. 2007). Both the model and the mimic species are rewarding, making this a case of mimicry of the Mullerian type, where the mimic gets a reproductive advantage due to increased visitation by bees when growing together with the more abundant model. Besides specific mimicry systems, convergence in flower colours might also arise among groups of co-flowering plants to improve overall recruitment of pollinators by forming pollination guilds (e.g. Schiestl and Johnson 2013).

**Fig. 5:**
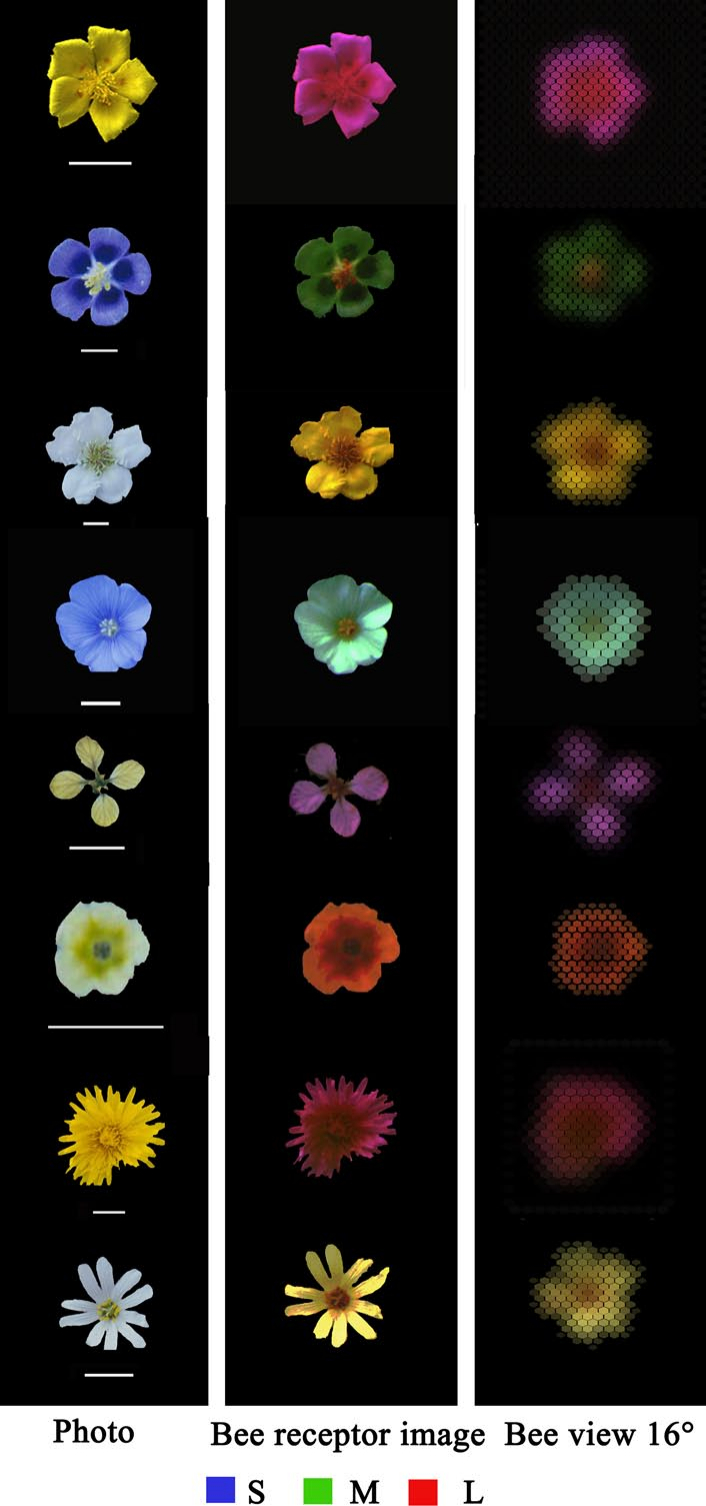
Flowers as seen through honeybee eyes. The figure shows displays (*human colours*) of flowers (1 cm scale). Flowers depicted are *Helianthemum nummularia*, *Aquilegia vulgaris*, *Mespilus germanica*, *Linum austriacum*, *Vella spinosa*, *Nonea lutea*, *Taraxacum officinale*, *Stellaria holostea*. Spectral sensitivities of the S, M, and L-receptors of honeybees were used to calculate quantum catches from multispectral images. To show ‘*bee colours*’ in the bee receptor images, we used the three primary colours of a computer monitor (*blue* for S, *green* for M and *red* for L). The* right column* shows the images of flower displays projected onto the ommatidial lattice of the bee eye (bee views), when they subtend 16° of visual angle, which is just above the chromatic threshold for detection and discrimination of coloured targets and patterns

Flowers may combine colours in their displays to form patterns or small ornaments, termed ‘nectar guides’. Spectral measurements of patterns are difficult to obtain with a photospectrometer due to its low spatial resolution, but multispectral imaging can be used for recording colours in flower patterns and reproducing how they look to the bee’s eyes (Vorobyev et al. 1997; Hempel de Ibarra and Vorobyev 2009; Fig. 5). Intact flowers and a standardised grey scale illuminated by diffuse natural daylight are photographed with a UV-sensitive CCD camera through five chromatic filters, which allows an accurate reconstruction of floral spectra (Vorobyev et al. 1997). These reflectance spectra are used to calculate the signals of the S-, M- and L-receptors in each pixel of the image, which are then converted into RGB values for displaying the image in ‘bee colours’ (Fig. 5). To simulate the optical resolution of the bee eye at different distances to the flower, the images are projected onto the ommatidial lattice and ommatidial quantum catches can be calculated (Vorobyev et al. 1997; Fig. 5).

**Fig. 6:**
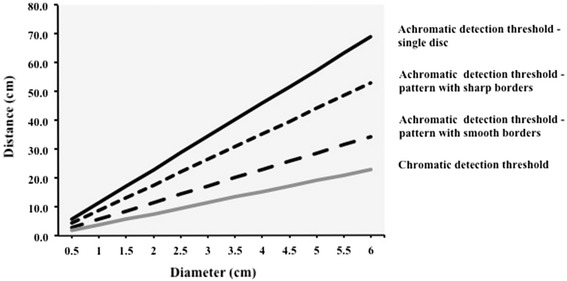
Detection ranges for coloured targets and concentric patterns of different sizes based on detection experiments with honeybees (Hempel de Ibarra et al. 2001). Patterns with smooth contrast borders are significantly impaired in detectability as compared to patterns with sharp borders or uniform L-contrast

Hempel de Ibarra and Vorobyev (2009) measured flowers displayed as single units from a wide range of wild bee-pollinated plant species across the angiosperm phylogeny. They simulated views seen at an angular size of 10°, which is within the operating range of the achromatic, L-receptor system that determines the maximal distance ranges for pattern detection in bees (Hempel de Ibarra et al. 2001). Quantum catches of ommatidia viewing the centre and the surround of the floral display were compared in flowers of different sizes. The spectral analysis evinced that small, individually displayed flowers with concentric patterns tend to exhibit a high-contrast outer ring. In behavioural experiments such patterns were detected by bees much better than patterns with smooth borders, where detectability is half that of single-coloured targets (Fig. 6). For small flowers, a suitable arrangement of L-contrast in their pattern could enhance the distance over which they are detected by bees, improving their chances when competing for pollinators against larger-sized flowers. It could be an adaptive trait that compensates for the small size of individual pattern displays (Hempel de Ibarra and Vorobyev 2009). One could argue that the effect might occur because the strength of L-contrast in the outer ring correlates with size. But neither the L-contrast in the ring nor in the central part of the pattern correlated with floral size. This result is in line with the findings of behavioural experiments, in which the detection limit did not depend on the strength of L-receptor contrast (Giurfa et al. 1996; Giurfa and Vorobyev 1998; Hempel de Ibarra et al. 2001). This study shows how flowers may benefit from evolving patterns that engage effectively with a bee’s visual pathways.

Linking the study of pollination systems with the quantitative assessment of floral features, from the point of view of the bee with its colour vision and learning abilities, has proved to be a very fruitful avenue for uncovering selective pressures that bees exert on flower colouration.

## Concluding remarks: colour vision and bee cognition

Advances in the study of colour vision and its neural mechanisms in insects would have been impossible without the rigorous implementation of psychophysical methods, characterisation of individual neurons and neural pathways, and modelling of experimental data in the honeybee. The successes in psychophysics and neurobiology of honeybee colour vision relied on behavioural procedures which limited the factors contributing to the response, namely colour discrimination. In the study of human colour vision the success of Newton, Young, Helmholtz, Maxwell, König, Hering and others was based on their focus on elementary forms of human colour discrimination under standardized conditions. Only then it was possible to formalise a concise theory of colour vision. Along the same logic, von Frisch and other bee vision researchers mentioned above targeted the elements of colour discrimination in honeybees by focusing on salient forms of learning. These forms of learning are based on simple discrimination tasks that are acquired quickly, such as training the animal to one colour signal under standardized conditions. Concise formal descriptions (i.e. models and hypotheses) of colour vision can be established only in this way. Additional forms of training leading to inhibitory learning (in the Pavlovian sense), or even combinations of reward learning and avoidance learning by punishment, were strictly avoided based on a simple argument: these complex forms of learning develop only in course of prolonged training protocols and lead to multiple memory traces corresponding to different behaviours. Studying such complex forms of learning with extended training protocols makes it impossible to capture the basic rules of colour discrimination in a formalised way and to establish quantitative relations to neural data. These studies have not led to any formal description and are bound to the phenomenal level.

Nevertheless, the interest in the learning abilities of honeybees is continuously growing, as it is a very amenable model system for the study of insect learning. A number of studies have demonstrated a remarkable flexibility in the algorithms that bees use to solve diverse sensory tasks. The focus of the emerging research has been on learning, but the intimate relationship between perceptual processes, learning and behavioural decision-making requires that each of the contributing processes is understood well. Often experiments rely on discrimination training of visual stimuli to show that the bee can solve a complex task presented by the experimenter, without considering what the bee perceives or how it might change its behaviour or learning strategy as the experimental protocol proceeds (see Stach and Giurfa 2005 for an example). Extended training protocols have to be carefully controlled and counterbalanced, avoiding unnecessary conditions, such as retraining the same bee to successions of different colours and shapes (e.g. Dyer and Neumeyer 2005; Dyer et al. 2008), and preventing uncontrolled behavioural biases or changes in behavioural rules. If bees rely on cues other than the hypothesised ones, invalid conclusions may be drawn. More elaborate stimuli and complex tasks bring with them a larger risk of offering unintended contingencies that can be exploited by bees, enabling them to come up with a different solution not necessarily involving the hypothesised mechanism.

Bee vision is inherently different from that of humans in many aspects. In studies where perceptual mechanisms are not clearly identified and stimuli are inspired by human vision rather than adopting the perceptual viewpoint of the bee, speculative classifications of visual representations or characterisation of visual stimuli as biologically relevant and irrelevant should be avoided. With regard to colour, this problem is exacerbated because stimuli may offer a range of cues to the bee, which involve both chromatic and achromatic mechanisms. Recently various studies have tested bees’ discrimination, generalisation or compound learning abilities in tasks with coloured scenes and complex patterns (e.g. Schubert et al. 2002; Zhang et al. 2004; Avarguès-Weber et al. 2012; Wu et al. 2013; for a discussion see Dyer 2012). When using a range of visual stimuli in training experiments it is usually simply assumed that they are perceptually dissimilar for bees (Benard et al. 2006). This assumption needs to be critically assessed and tested, which is rarely done whilst being crucial for discussions about the nature of stimulus representations and the complexity of any involved mechanisms. Understanding perceptual similarities between coloured stimuli, patterns or scenes for bees is important, so accurate quantification of chromatic and achromatic cues (including UV) and testing for variable use of cues across different task settings must be encouraged. Although complex test conditions provide us with interesting data, they may not be suitable for formal descriptions attempting to model the basic psychophysical rules of colour discrimination, and obviously new approaches are required.

The honeybee is an excellent model system to investigate colour vision at a number of levels, from perception and neural mechanisms to ecology and evolution. A solid knowledge base has been established, and more details are to be revealed as we embark upon the second century of honeybee vision research.

## Appendix

### Modelling colour discrimination in the honeybee

Colour can be described by photoreceptor quantum catches, *Q*_*i*_, which are calculated as:

1 $$Q_i = C_i \int {I (\lambda )R_i (\lambda )d\lambda } ,$$

where *i* = *S*, *M*, *L* denotes the spectral type of a photoreceptor, *λ* is the wavelength, *I* ^*e*^(λ) is the spectrum of the light entering the eye, *R*_*i*_(λ) is the relative spectral sensitivity of a receptor of a spectral type *i*, and *C*_*i*_ the scaling factors (constants) describing absolute sensitivity of photoreceptors. Quantum catches are measured as the number of absorbed photons per integration time. Because the illumination spectrum is given in number of photons/(nm m^2^ radian^2^ s^2^) the units for scaling factors *C*_*i*_ are m^2^ radian^2^ s^2^.

Of practical importance is the case when surface colours are considered. The spectrum of the light reflected from a surface with spectral reflectance, *S*(λ), illuminated by a light source with spectrum *I*(λ) equals to *I*(λ)*S*(λ). Therefore, for a visual system viewing a surface with spectral reflectance *S*(λ) illuminated by a light source with spectrum *I*(λ), photoreceptor quantum catches are given by

2 $$Q_i^w = C_i \int {I (\lambda )S(\lambda )R_i (\lambda )d\lambda } .$$

Instead of absolute quantum catches, often relative quantum catches, *q*_*i*_, are used

3 $$q_i = \frac{Q_i }{Q_i^w }$$

where, *Q*_*i*_^*w*^ = *C*_*i*_ ∫ *I*(*λ*)*R*_*i*_(*λ*)*dλ* are quantum catches describing colour of illumination, i.e. quantum catches corresponding to an ideal white surface. Note that relative quantum catches, *q*_i_, do not depend on scaling factors, *C*_*i*_. Relative quantum catches remain largely invariant in conditions of changing illumination and Eq. (14) describes von Kries colour constancy.

The first theory of colour vision has been proposed by Backhaus (1991) on the basis of multidimensional scaling of the honeybee colour choices. He assumed that receptor signals can be described by ‘excitations’:

4 $$E_i = \frac{q_i }{1 + q_i }$$

Coding is performed by two colour-opponent mechanisms termed *A* and *B*, whose outputs are calculated as:

$$A = - 9. 8 6E_s + 7. 70E_b + 2. 1 6E_L$$

5 $$B = - 5. 1 7E_s + 20. 2 5E_b - 1 5.0 8E_L$$

The axis corresponding to direction A and B are assumed to form ‘perceptual colour’ space of a honeybee. The distance in this space is calculated using city-block metric as

6 $$\Delta S = |A| + |B|$$

The model describes a large body of behavioural data (Vorobyev and Brandt 1997). It is important to note that the proposed ‘perceptual colour space’ does not correspond to chromatic diagram, i.e. the position of a light stimulus in this space depends on its intensity. Therefore the model incorrectly predicts that colour discrimination deteriorates as the intensity of light stimuli increases (Hempel de Ibarra et al. 2000; Vorobyev et al. 1999). In particular, the model predicts that bees are not able to discriminate bright UV-reflecting white flowers, from green leaves. This prediction has been shown to be incorrect, as bees can easily discriminate bright UV-reflecting white from leaf-like green (Hempel de Ibarra et al. 2000; Vorobyev et al. 1999).

A different approach to modelling is based on the assumption that the noise originating in photoreceptors limits colour discrimination. Photoreceptors are biological light-measuring devices. For a light-measuring device with no internal noise, the error is determined solely by fluctuations of the number of absorbed photons per integration time. The number of absorbed photons has a Poisson distribution and therefore the fluctuations (standard deviation) of the number of absorbed quanta, *δ* *Q*_*i*_^*R*^, are equal to the square root of the number of absorbed quanta (Rose–de Vries law):

7 $$\delta Q_i^R = \sqrt {Q_i }$$

In addition to the fluctuations of the number of absorbed quanta, other sources of noise affect the accuracy of measuring the light (Vorobyev et al. 2001a). At very low light levels, the dark noise, *d*_*i*_, plays important role. This noise does not depend on the number of absorbed photons:

8 $$\delta Q_i^d = d_i .$$

As the intensity of illumination increases the noise increases faster than Eq. 3 predicts. This noise can be described by Weber law, which states that the noise is proportional to the signal, i,e.

9 $$\delta Q_i^w = w_i Q_i ,$$

where, *ω*_*i*_ is equivalent to Weber fraction. Combination of the three sources of noise gives:

10 $${\delta Q_i = \sqrt {d^2 + Q_i + \omega_i^2 } Q_i^2 }$$

The relative noise is defines as

11 $$\frac{\delta Q_i }{Q_i } = \sqrt {\frac{d^2 }{Q_i^3 }d^2 + \frac{1}{Q} + \omega_i^2 }$$

The relative noise can be decreased by summation of signals of *n* photoreceptor cells by a factor $(\sqrt n)$.

The Weber law gives a fair approximation of the noise to signal ratio over a wide range of light intensities because photoreceptors adapt to changing illumination conditions (for review see Osorio and Vorobyev 2005). The Weber law implies that relative values of the receptor quantum catches, *q*_*i*_, are sufficient to describe the noise in receptor channels. When the Weber law is valid it is practical to describe photoreceptor signals, *f*_i_, using logarithmic transformation of quantum catches

12 $$f_i = { \ln }({q_i }) = { \ln }({Q_i }) - { \ln }(Q_i^w )$$

From Eqs. 9 and 12, it follows that the variation of receptor signal so defined is equal to the Weber fraction because

13 $$\delta f_i = \frac{df_i }{dq_i }\delta q_i = \frac{{d\;{\text{ln(}}q_i )}}{dq_i }\omega_i q_i = \frac{\omega_i q_i }{q_i } = \omega_i$$

Colour can be represented as a point in colour space where the separation of any two points in this space can be assigned a distance, Δ*S*. When the distance is less than a certain threshold distance, Δ*S* ^*t*^, the stimuli are not discernable. The receptor noise-limited colour-opponent model (Vorobyev and Osorio 1998; Vorobyev et al. 2001a) is based on the following assumptions.

1. In a colour vision system with three spectral types of photoreceptors, colour is coded by at least two unspecified colour-opponent mechanisms.

2. Colour-opponent mechanisms are not sensitive to changes in the stimulus intensity.

3. The sensitivity of these mechanisms is set by noise originating in photoreceptors.

Let *f*_i_ be receptor signals scaled so that the increase of the intensity of a light stimulus adds the same value to all-receptor signals (e.g. *f*_*i*_ = ln(*q*_*i*_), see Eq. 12), *ω*_*i*_ be the noise in the receptor mechanisms and ∆*f*_i_ be the difference in receptor signals between two stimuli, then the distance between two colour stimuli can be calculated as

14 $$\Delta S = \sqrt {\frac{\omega_s^2 (\varDelta f_L - \varDelta f_M )^2 + \omega_m^2 (\varDelta f_L - \varDelta f_s ) + \omega_L^2 (\varDelta f_M - \varDelta f_S )^2 }{\omega_L^2 \omega_M^2 + \omega_L^2 \omega_S^2 + \omega_M^2 \omega_S^2 }}$$

Note that when Weber law applies all *ω*_*i*_ remain constant. Electrophysiological measurements of the signal-to-noise ratio in the honeybee photoreceptors give the following results: *ω*_*S*_ = 0.13, *ω*_*M*_ = 0.06, *ω*_*L*_ = 0.12 (Vorobyev et al. 2001a). Predictions based on Eq. 14 are in excellent agreement with behavioural data (Vorobyev et al. 2001a).

Equation 14 describes distance in chromatic plane. The following transformation of receptor signals allow to construct an equal distance colour space, where Euclidean distance corresponds to the distance given by Eq. 14 (Kelber et al. 2003):

15 $$\begin{gathered} X_1 = A(f_L - f_M ) \hfill \\ X_2 = (Bf_S - (af_L + bf_M )), \hfill \\ \end{gathered}$$

where

$$\begin{gathered} A = \sqrt {\frac{1}{\omega_L^2 + \omega_M^2 }} \hfill \\ B = \sqrt {\frac{\omega_L^2 + \omega_M^2 }{\omega_L^2 \omega_M^2 + \omega_L^2 \omega_S^2 + \omega_M^2 \omega_S^2 }.} \hfill \\ a = \frac{\omega_M^2 }{\omega_L^2 + \omega_M^2 } \hfill \\ b = \frac{\omega_L^2 }{\omega_L^2 + \omega_M^2 } \hfill \\ \end{gathered}$$

## Abbreviations

COC: Colour-Opponent Coding model
RNL: Receptor Noise-Limited Colour-Opponent model
S-/M-/L-receptor: Short-/middle-/long-wavelength-sensitive photoreceptor
